# Responding to the HIV Health Literacy Needs of Clients in Substance Use Treatment: The Role of Universal PrEP Education in HIV Health and Prevention

**DOI:** 10.3390/ijerph20196893

**Published:** 2023-10-07

**Authors:** Yusen Zhai, Kyesha M. Isadore, Lauren Parker, Jeremy Sandberg

**Affiliations:** 1 Department of Human Studies, The University of Alabama at Birmingham, Birmingham, AL 35294, USA; 2 Department of Rehabilitation Psychology and Special Education, University of Wisconsin-Madison, Madison, WI 53706, USA; isadore@wisc.edu; 3 Department of Educational Psychology, Counseling, Special Education, College of Education, The Pennsylvania State University, University Park, PA 16802, USA; ldp125@psu.edu (L.P.); jts32@psu.edu (J.S.)

**Keywords:** pre-exposure prophylaxis (PrEP) education, PrEP health literacy, substance use disorders, HIV prevention, counselors

## Abstract

Health literacy, particularly HIV health literacy, is a key social determinant of health and can be significantly improved through targeted health education. This paper explores the often-overlooked potential of pre-exposure prophylaxis (PrEP) education as a powerful tool to enhance HIV health literacy among people with substance use disorders (PWSUD), a population notably susceptible to HIV. Given the syndemic interplay of substance use disorders (SUDs) and HIV, health professionals, especially substance use counselors, are uniquely positioned to bolster HIV health literacy and positively influence health outcomes. This article offers a brief introduction to PrEP, delineates potential barriers and facilitators to its use and education, and proposes strategies for effective PrEP education, implementation, and adherence. By equipping substance use counselors with essential knowledge and skills, we aim to encourage and promote the integration of PrEP education into substance use treatment. The overarching objective is to empower counselors to proactively engage in HIV prevention efforts, thereby fulfilling pressing health literacy needs and contributing to improved health outcomes among PWSUD.

## 1. Introduction

According to the U.S. Department of Health and Human Services [1], health literacy is a key social determinant of health that is linked with a range of health outcomes. People with poor personal HIV health literacy tend to be less likely to use preventive measures, such as pre-exposure prophylaxis for HIV (PrEP), and thus are less able to successfully prevent the transmission of HIV [2]. Similarly, substance use treatment providers with poor organizational HIV health literacy tend to be less likely to provide clear and comprehensible information about HIV and preventive measures, including PrEP, and thus are less able to prevent HIV transmission among people with substance use disorders (PWSUD). PrEP is a preventive strategy wherein HIV-negative individuals who are susceptible to HIV acquisition take specific anti-HIV medications on an ongoing basis, or as needed, to greatly reduce their risk of acquiring HIV. When taken consistently, PrEP can reduce HIV acquisition from sex by over 90% and from intravenous drug use by more than 70%, making it a highly effective tool in HIV prevention [3]. However, among those who would likely benefit from PrEP, many lack awareness of what PrEP is [4]. PrEP education promises to be a powerful tool to improve HIV prevention and health outcomes through increased PrEP utilization amid the syndemic of substance use disorders (SUDs) and HIV.

The concept of a “syndemic” refers to the synergistic interaction of multiple diseases within a population, compounded by social, environmental, and economic factors [5,6]. A stark example of this phenomenon is observed in the intertwined epidemics of SUDs and infectious diseases, such as HIV, sexually transmitted infections (STIs), and viral hepatitis, which collectively exacerbate the health burden on affected populations [4,5]. Particularly, PWSUD present heightened vulnerability to HIV, STIs, and hepatitis [4,5]. This susceptibility stems from a confluence of social determinants of health, including unsafe injection practices, sexual behaviors often linked with substance misuse, limited access to healthcare services, and a plethora of socioeconomic hardships such as poverty, housing instability, and incarceration [4,5]. A syndemic perspective thus underscores the intricate biological, behavioral, social, and structural dynamics at play, suggesting the need for comprehensive, integrated approaches in the prevention and treatment of these interconnected health issues. Hence, the integration of universal PrEP education in substance use treatment has emerged as an essential component of a comprehensive, integrated approach to address the syndemic through efforts in HIV prevention [7].

The U.S. National HIV/AIDS Strategy (NHAS) and the National HIV/AIDS Strategy Implementation Plan (NHASIP) have highlighted the importance of integrating services to effectively address the syndemic of HIV and SUDs [8]. Specifically, they aim to prevent new HIV infections by improving HIV health literacy, including increased awareness of HIV and its prevention (e.g., PrEP) and integrated HIV messaging [7]. This strategy aligns with the recent revisions in the U.S. Centers for Disease Control and Prevention guidelines [7], which now advocate for the provision of PrEP education to all sexually active adolescents and adults as well as persons who inject drugs. While the interconnected factors and comorbid conditions impacting the syndemic of HIV and SUD require comprehensive strategies to address the multiple complexities of this health issue, the primary focus of this article is on the prevention of HIV through increased PrEP education.

In this article, we aim to address the need for substance use counselors to provide universal PrEP education as a key intervention to improve HIV health literacy and reduce HIV transmission among PWSUD. According to the Substance Abuse and Mental Health Services Administration [9], all substance use counselors must acquire the knowledge of HIV and its prevention and develop skills in providing targeted education to improve HIV health literacy, ultimately contributing to promotion and maintenance of health. Additionally, the current NHASIP [8] and CDC guidelines [7] underscore the importance of expanding access to PrEP education. Yet, there has been a lack of organizational HIV health literacy among substance use counselors due to a persistent knowledge gap among many counselors. Also, many counselors are not aware of the CDC guidelines that recommend universal PrEP education for all people who are sexually active or who inject drugs. To bridge the gap, this article provides key information and skills needed for substance use counselors to improve organizational HIV health literacy so that they can take a more proactive role in HIV prevention efforts and personal HIV health literacy promotion by providing their clients with education on HIV and PrEP. The following topics will be addressed: (a) the role of PrEP education in improving HIV health literacy as a key social determinant of HIV health and prevention, (b) an overview of PrEP for substance use counselors, (c) barriers and facilitators to PrEP use and education, and (d) practical strategies to facilitate PrEP education, implementation, and adherence.

## 2. HIV Health Literacy as a Social Determinant of HIV Health and Prevention

Substance use counselors constitute a critical component of the professional workforce in enhancing health outcomes among PWSUD. A fundamental strategy in achieving this is that of bolstering HIV health literacy, a key social determinant of HIV health and prevention [10,11]. Personal health literacy encapsulates the ability of an individual to “find, understand, and use” health-related information and services, such as PrEP, for oneself and others [12]. Concurrently, organizational health literacy concerns the degree to which organizations, inclusive of health professionals, equitably enable individuals in this process. The synergy between personal and organizational health literacy underscores the need for a holistic approach to HIV health literacy, which encompasses enhancing awareness and knowledge about HIV preventive measures, such as PrEP, among substance use counselors and their clients [13,14]. The NHAS has called for strategies to address the syndemic of HIV, SUDs, STIs, and viral hepatitis, which underscores the critical role that substance use counselors can play in integrating HIV prevention and education in their practice.

Substance use counselors serve PWSUD who are uniquely vulnerable to HIV exposure, yet a growing body of literature indicates that many substance use counselors and health professionals are not aware of PrEP [15,16,17,18]. Likewise, PWSUD have limited knowledge of PrEP and awareness of PrEP accessibility [4,19,20]. These limitations in organizational and personal HIV health literacy regarding PrEP produce significant barriers and pose a threat to health outcomes among PWSUD. PWSUD have a heightened vulnerability to HIV transmission if they share syringes, while intoxication caused by substance use can impair judgment and increase the possibility of engaging in condomless sex, thereby increasing the likelihood of acquiring HIV sexually. These transmissions are particularly concerning within a substance use disorder context as they can lead to outbreaks of HIV among social networks of PWSUD [21]. Such outbreaks jeopardize the lives of PWSUD, underscoring the urgency of PrEP education and use to increase HIV health literacy and prevent HIV among individuals with a greater likelihood of direct exposure.

Existing research posits that interventions aiming to enhance health literacy can function as midstream strategies to promote health outcomes and address health disparities, given the integral role of health literacy as a key social determinant of health [2,11]. Nutbeam and Lloyd, for instance, contend that health literacy can be augmented through high-quality, context-specific education [1]. Within the domain of HIV health and prevention, universal PrEP education thus emerges as a midstream intervention to bolster HIV health literacy, contributing to improved health outcomes and the amelioration of disparities among PWSUD with diverse marginalized identities.

## 3. Overview of PrEP for Substance Use Counselors

For substance use counselors, understanding how HIV compromises the immune system and how PrEP works at a fundamental level is essential for improving providers’ organizational HIV health literacy. With increased organizational HIV health literacy, counselors can educate their clients about HIV and PrEP to improve personal HIV health literacy effectively.

HIV, a retrovirus, primarily targets CD4+ immune cells. Once inside CD4+ cells, HIV hijacks the cell machinery to replicate HIV rather than to protect the body from illness. As such, HIV leads to a weakened immune system that, untreated, leaves the body vulnerable to opportunistic infections and eventually death. The HIV replication process that takes place in CD4 cells can be broken down into seven steps: (1) HIV binding to CD4 cell receptors; (2) membrane fusion, allowing HIV entry into the CD4 cell; (3) reverse transcription, converting HIV RNA to DNA; (4) integration of HIV DNA with CD4 cell DNA; (5) replication of the necessary protein chains; (6) assembly of new HIV proteins and RNA; and (7) budding, wherein HIV exits the CD4 cell and matures to eventually repeat this process with another CD4 cell. Importantly, primary HIV infection does not occur until the fourth step, integration, after an incubation period of 12 to 72 h from initial exposure [22,23].

PrEP is a biomedical intervention (i.e., medicine) for HIV prevention that emerged from the successful use of antiretroviral therapy (ART) to control HIV replication in people living with HIV (PLHIV). PrEP can be taken as a pill or via injection and is able to prevent HIV even if exposure occurs. PrEP interrupts the HIV replication process before step four (i.e., infection) described above, meaning that even if exposure occurs, PrEP can prevent HIV infection at the cellular level, and a person will remain negative for HIV. Studies have demonstrated that consistent PrEP use reduces HIV acquisition by over 90% for sexual contact and more than 70% for injection drug use [3]. This said, it is important for counselors to know and inform their clients that PrEP is not sufficient for treating HIV, as it only contains some of the medications that are required to treat someone living with HIV.

Since 2012, the FDA has approved three PrEP medications: emtricitabine and tenofovir disoproxil fumarate (F/TDF), emtricitabine and tenofovir alafenamide (F/TAF), and cabotegravir extended-release injectable suspension (CAB). The U.S. Preventive Services Taskforce awarded an “A” rating for PrEP use in 2019, supporting its effectiveness and increasing the likelihood of insurance coverage [24]. The 2021 updated U.S. Public Health Services PrEP guidelines recommend that counselors and drug and alcohol treatment providers inform all sexually active clients/patients and persons who inject drugs about PrEP and its role in preventing HIV [7]. While PrEP is a powerful tool for HIV prevention, various barriers can hinder its implementation, use, and education. This highlights the importance of substance use counselors being equipped with comprehensive knowledge about such barriers so that they can effectively address these barriers and facilitate PrEP use and education to improve HIV health literacy, reduce HIV transmission, and contribute to better health outcomes among PWSUD.

## 4. Barriers to PrEP Use and Education

Although the development of PrEP (in addition to the use of ART among PLHIV), provides a biomedical pathway to end the HIV epidemic, HIV incidence has only decreased by about 8% from 2015–2019 [7]. The FDA approved PrEP in 2012, yet only about 30% of the 1.2 million people with an indication for PrEP received a PrEP prescription in 2021 [7,25]. Although PrEP is underutilized by all groups, greater disparities in PrEP uptake are evident among racial/ethnic minorities, people from rural communities, and adolescents and young adults [19,25]. In particular, people who experience socioeconomic inequalities and have limited access to health care, health education, and preventative medicine have the lowest levels of PrEP uptake [20,26]. The varied utilization of PrEP among different sociodemographic groups appears to be a reflection of systemic constructs and barriers highlighted by the social determinants of health [27].

Considering these disparities, substance use counselors can work to advocate for clients within these communities and tailor PrEP education to meet their cultural and health literacy needs [14]. Despite the potential of PrEP education to enhance HIV health literacy and increase PrEP access among some PWSUD, barriers persist that may deter these individuals from accessing PrEP or articulating their need for it. Substance use counselors should be equipped to inform clients not only about the nature of PrEP but also about resources that can facilitate access to PrEP, such as insurance and other forms of funding. For clients who are hesitant or unable to express their need for HIV preventive measures like PrEP, counselors must consider the influence of stigma and trauma associated with healthcare systems and HIV [13]. Many barriers identified by the social determinants of health framework contribute to the persistence of disparities in PrEP uptake, including the lack of client and provider HIV health literacy (e.g., knowledge and comfort in HIV preventions), financial and geographical barriers, housing instability, and stigma.

### 4.1. Lack of HIV Health Literacy

#### Personal HIV Health Literacy

A primary barrier to PrEP utilization is the lack of personal HIV health literacy; many people in need of PrEP do not know it exists [15,28]. There is some variability in outcomes related to levels of PrEP awareness and knowledge, with some studies highlighting recent increases in PrEP awareness, while others suggest low levels of PrEP knowledge among various groups [4,19,28,29,30,31,32]. Lack of knowledge about PrEP might contribute to HIV health disparities because individuals are not able to take PrEP if they do not know it exists. Overall, research indicates that knowledge of PrEP is lower among racial/ethnic minorities and in specific geographical regions such as rural communities and the southern region of the United States [19,33,34]. One study centered in rural Appalachia found that 68.8% of participants—all of whom had a history of injection drug use—had never heard of PrEP, and none of those who had were able to accurately describe its purpose for HIV prevention [19].

### 4.2. Organizational HIV Health Literacy

A key barrier to improving PWSUD’s personal HIV health literacy and implementing PrEP education is the concurrent meager organizational HIV health literacy. For example, there has been a lack of knowledge about PrEP among many medical and substance use treatment providers. Despite increasing awareness and knowledge of PrEP over the past few years, Sell et al. found that more PrEP-related discussions are initiated by clients than providers (54% vs. 39%) in rural and suburban areas [35]. As such, substance use counselors cannot rely on primary care providers to offer PrEP education; counselors should be able to deliver PrEP education to improve the personal HIV health literacy and outcomes of their clients. To this end, counselors must have knowledge of HIV and related preventive measures, such as PrEP, to improve organizational HIV health literacy. However, inadequate organizational HIV health literacy has become a barrier for substance use counselors in delivering PrEP education. For instance, substance use counselors may not have access to up-to-date information about PrEP and related resources and lack awareness of barriers to PrEP use and education, which may deter them from implementing the most recent CDC recommendations to provide all sexually active adolescents and adults as well as persons who inject drugs with universal PrEP education and facilitate referral to PrEP care for those who could benefit from biomedical HIV prevention interventions [36].

In fact, substance use counselors may be a strong workforce offering universal PrEP education because they are adept in navigating stigma and addressing the intersecting barriers PWSUD face, which makes them uniquely positioned to increase PWSUD’s personal HIV health literacy, including PrEP knowledge and access. With increased organizational HIV health literacy, such as knowledge and comfort in PrEP related discussions, substance use counselors are strong candidates to provide universal PrEP education and respond to the specific needs shared by their clientele [19,34].

Increasing substance use counselors’ understanding of the personal HIV health literacy needs of PWSUD is a critical step forward toward improving organizational HIV health literacy to better structure and deliver universal PrEP education. This requires acquiring comprehensive knowledge about PrEP and its accessibility. Improvements in organizational HIV health literacy among substance use counselors will enable them to harness PrEP education as a midstream intervention to address health disparities and bolster personal HIV health literacy among PWSUD disproportionately impacted by the syndemic of HIV and SUD. It is anticipated that PWSUD with better HIV health literacy will be more likely to use HIV preventive measures, including PrEP, and will be more able to successfully prevent HIV transmission [2]. Given the potential influence of PrEP education on HIV health and prevention, counselors must acquire awareness and knowledge of the additional barriers to PrEP use and education, as well as the ways in which recent advancements can aid in overcoming these hurdles in the future.

### 4.3. Financial Barriers

A key component of HIV health literacy is the ability to access and utilize HIV preventive resources to inform health decisions and actions [1], which emphasizes the urgent need to address barriers to financial literacy regarding PrEP utilization. Studies demonstrate that both the actual and perceived costs of PrEP are barriers for those in need of PrEP [19,33,34]. Many individuals considering PrEP have heard that PrEP is expensive for those who are uninsured, and may still be costly for those who are insured [34]. Some individuals currently on PrEP or who have attempted PrEP initiation have reported challenges affording both doctor copays and medication costs [19], as well as additional financial barriers to the PrEP continuum when their insurance does not cover the routine HIV testing or follow-up appointments [33].

According to Neilan et al. [37], the cost of current PrEP medications varies widely; however, insurance should cover the cost of more expensive medications if the need is medically justified. In addition to the prescription costs associated with PrEP, other expenditures include medical visits, transportation to and from medical visits, and time away from work when following PrEP protocols [15,19,33]. Recent developments have cast uncertainty on the Affordable Care Act’s (ACA) mandate, which previously stipulated that health insurance companies provide medical services with a Grade A recommendation from the U.S. Preventive Services Task Force (USPSTF), without necessitating cost-sharing. A federal ruling has now contested the coverage of certain preventive health services and medications, including PrEP.

The relationship between a person’s socioeconomic status and their decision to use PrEP is influenced by their level of HIV health literacy [38]. USPSTF recommendations have improved some of the opportunities to access PrEP since January of 2021, and counselors should share updated information and resources to inform their clients about their options for PrEP. In fact, updated CDC PrEP guidelines specifically state that providers should offer benefits counseling to assist eligible clients in obtaining insurance [7]. To do this, counselors need to be aware of key resources that can help to reduce the costs of PrEP care, such as “Ready, Set, PrEP”, which makes PrEP medications available at no cost via the website www.readysetprep.hiv.gov (accessed on 21 July 2022). Additional information about state-specific resources to assist with PrEP costs can be found at https://nastad.org/prepcost-resources/prep-assistance-programs (accessed on 21 July 2022).

Despite these provisions, health professionals may not be aware of these changes and the options available to make PrEP and associated costs affordable. Furthermore, the time, effort, and knowledge required to navigate this system may form an additional barrier to PrEP access. Thus, substance use counselors should integrate information on financial assistance into PrEP education. Increasing knowledge of the financial hardship clients may experience in navigating healthcare systems and awareness of key financial resources can help substance use counselors to educate clients, advocate for their clients, and connect them to quality care.

### 4.4. Geographical Barriers

While PrEP has demonstrated significant potential in preventing people with increased exposure to HIV from acquiring HIV, its implementation, use, and education has been hindered by various geographical barriers such as healthcare accessibility, the distribution of healthcare facilities, and rural–urban disparities. For example, healthcare capacity is far less adequate (i.e., there are fewer HIV specialists and PCPs per capita) in the southern region of the United States [13]. This region, in which 37% of the total U.S. population lives, is home to 55% of the Black/African American population [39]. This has led to not only regional disparities in PrEP education and uptake, but also significant racial disparities. Furthermore, PrEP services are less likely to exist in rural communities, which may cause difficulties for some individuals in accessing PrEP and PrEP education (e.g., there are only 324 known PrEP users in the state of West Virginia [19]). To combat geographical barriers to PrEP, access could be integrated within other services that PWID may already frequent, such as syringe support programs and drug and alcohol treatment facilities [18]. Also suggested is that PrEP supplies (e.g., daily pills) should be distributed at locations frequently visited by PWID. Furthermore, the utilization of telehealth services and mobile clinics can help overcome geographical barriers by providing virtual consultation, prescription refills, and remote monitoring for PrEP users [40]. Substance use counselors should inform clients about these services, which can expand access to PrEP for clients with these geographical barriers or those with limited transportation options.

### 4.5. Housing Instability

Housing instability, another social determinant of health, serves as another barrier which can significantly impact PrEP education and the implementation and use of PrEP [41,42]. In a study by Allen et al. [19], some participants who were experiencing homelessness or who were unstably housed believed this would be their biggest hurdle for utilizing PrEP, and that the goal of utilizing PrEP would be overridden by their immediate priorities (e.g., finding a safe space to sleep). Other participants identified the lack of secure storage for their medicine as a significant and insurmountable impediment to sustained PrEP use, as they felt their medication would be stolen or damaged from rain and snow [18]. Overall, health literacy is lower amongst people with vulnerable or unstable housing [43,44]. To support clients with housing instability, counselors should educate their clients about safe storage opportunities, where clients can securely store their medication or other PrEP supplies. This may include local organizations that provide lockers or trusted shelters. Additionally, counselors can educate their clients about local integrated care centers where HIV prevention services are combined with other essential services for individuals experiencing housing instability, including financial assistance programs and supportive housing programs. Furthermore, Gregg et al. [43] identified inconsistent screening by clinic staff in Veterans Affairs settings as a barrier to accessing PrEP for unstably housed veterans. Thus, substance use counselors working with clients through the Veterans Affairs system who may have unstable housing should inform clients about their eligibility to receive PrEP, and empower clients to self-advocate if encountering inconsistent screening procedures.

### 4.6. Stigma

A review of the research literature reveals that stigma—a structural-level barrier and social determinant of health—informs and permeates disparities in healthcare access and associated client and provider barriers to PrEP education, access, and care. HIV is highly stigmatized, and PrEP use is also stigmatized due to its connection to HIV susceptibility. Despite 20–30% of PLHIV being heterosexual, many people associate PrEP with being gay. Many also associate HIV with using drugs, having multiple sexual partners, and irresponsible behavior [13,34]. Due to HIV stigma, HIV has long been a taboo subject, which prevents many individuals from accessing HIV information. Additionally, many perceive that HIV is no longer a major concern, and may not be aware of their risk [34]. Some cultural and social norms silence discussions concerning sexuality, which creates barriers to PrEP knowledge dissemination. These norms, along with fear of judgment, negatively affect the level of encouragement and other social support that individuals may receive from others around the use of PrEP [33]. Social norms and stigma around sexuality also pervade healthcare settings providing PrEP [33], which creates additional barriers once individuals enter those settings. Moreover, participants from several studies reported experiencing PrEP-related stigma that manifested in various ways, including stereotyping, rejection, and discrimination, all of which can be considered barriers to PrEP use [15].

Stigma can also lead to distrust of healthcare providers. Participants in a qualitative study of queer and transgender adults discussed how negative relationships with their providers impeded their access to PrEP [45]. Specifically, providers who demonstrate discomfort with sexuality by not discussing sexual history, not offering PrEP to clients who meet the criteria, using stigmatizing language, and not offering risk reduction or PrEP adherence counseling, have created a challenging environment in which clients can receive knowledge or initiate PrEP. Another study highlighted participants’ distrust of healthcare providers, particularly among populations with disproportionate HIV vulnerability who experienced alienation from the healthcare system because of “experienced and anticipated discriminatory judgment from providers in the form of racism or homophobia” [15] (p. 1790). Subsequently, distrust of healthcare providers has become one of the prominent barriers to PrEP access among people susceptible to HIV.

Social and internal stigma surrounding HIV continues to deter people who might be vulnerable from seeking HIV prevention treatment [12,33]. Some individuals might fear being ostracized from their community if it was discovered that they were taking PrEP, and others might fear being subject to prejudice and discrimination from other people, including health professionals [12,33]. Substance use counselors, given their training, are equipped to manage sensitive dialogues, such as those related to HIV health and prevention, employing a person-centered and affirming approach [46]. With the amplification of their knowledge and awareness concerning HIV health and prevention, these counselors have the capacity to critically evaluate their attitudes towards HIV, thereby nurturing a supportive and non-judgmental environment [9]. This environment enables the validation of clients’ apprehensions regarding HIV and assists in the process of addressing the clients’ internalized stigma associated with HIV and its prevention [47]. The establishment of a robust therapeutic alliance and a level of comfort in discussing HIV and prevention can lay the groundwork for delivering universal PrEP education. This can contribute significantly to enhancing HIV health literacy among PWSUD.

In addition to internalized stigma, clients may encounter external discrimination in the form of provider bias. It is critical for counselors to comprehend how discussions about PrEP with clients susceptible to HIV might inadvertently activate their own preconceived notions. Hence, a proactive approach in seeking consultation, receiving guidance from other professionals, and participating in continual professional development through training and education is paramount to ensure the provision of the highest level of care. A prevailing assumption persists within society, suggesting that individuals susceptible to HIV are consciously choosing to expose themselves to risk, and that a positive HIV test result is the sole fault of the individual [48]. This stereotype underpins the importance of substance use counselors challenging and reevaluating their own biases. Mitigating these biases is integral to reducing the spread of HIV and minimizing the risk of transmission. A nuanced understanding of the potential for bias is particularly important given that individuals susceptible to (e.g., PWSUD) or living with HIV may be less likely to seek support or disclose their status if they harbor fear of prejudice and discrimination. Therefore, fostering an environment free of bias and judgment is essential in encouraging open dialogue on HIV prevention and improving health literacy and outcomes among clients with substance use disorders.

## 5. Recommendations for Clinical Practice to Deliver PrEP Education and Implementation within a Trauma-Informed Framework

### 5.1. A Trauma-Informed Universal PrEP Education Framework

Given the high prevalence of trauma, which is known to impact people seeking substance use treatment [49], we have developed a trauma-informed framework that integrates the CDC’s guidelines regarding universal PrEP education for this vulnerable population [7]. This integrated framework aims to guide substance use counselors to address the growing HIV health literacy needs and barriers to HIV prevention experienced by clients in substance use treatment. With the CDC’s recommendation of universal PrEP education for all sexually active adolescents and adults as well as persons who inject drugs, substance use counselors have a unique opportunity to reduce stigma and increase clients’ HIV health literacy through trauma-informed universal PrEP education. By adopting and implementing this integrated framework, substance use counselors will be better able to understand and process trauma and its effects, particularly at the intersection with substance use and HIV risk, and will be able to create a supportive, non-judgmental, and respectful environment that feels safe to clients [50]. Substance use counselors can collaborate with their clients in decision-making and treatment/referral planning during PrEP education, which encourages clients to take an active role in making their own informed decisions based on the information provided by counselors. This approach can help clients regain control often lost in traumatic experiences [51] by empowering them to proactively manage their susceptibility to HIV through the use of PrEP. Utilizing PrEP as a preventive measure offers PWSUD the tool to reduce their likelihood of acquiring HIV, thereby fostering autonomy and control over their health. Lastly, practicing cultural humility by respecting the diverse cultural backgrounds of clients can influence attitudes towards HIV, PrEP, and healthcare in general [52]. By integrating trauma-informed approach into daily practice, substance use counselors can help their clients feel more understood and supported, and feel more receptive to PrEP education, ultimately leading to better health literacy and health outcomes.

### 5.2. Integrating Universal PrEP Education within Trauma-Informed Care

As noted throughout this article, lack of HIV health literacy is a key social determinant of HIV health and prevention that needs greater attention within substance use counseling communities. Integrating universal PrEP education within a trauma-informed framework can be a critical step forward to promoting HIV health literacy and reducing HIV among PWSUD. The NHASIP and the CDC encourage substance use counselors to incorporate PrEP education as part of routine care for all sexually active clients and persons who inject drugs. Given the benefit of trauma-informed care, substance use counselors can incorporate the proposed trauma-informed PrEP education into an initial intake or level-of-care assessment. To increase awareness of PrEP, counselors may also display PrEP materials through social media posts, educational posters, and multi-lingual pamphlets, using respectful and culturally sensitive language.

Utilizing a trauma-informed framework entails creating a safe and non-judgmental space for clients, ensuring that educational content and methods are sensitive to potential triggers related to trauma histories and personal experiences. We recommend the following script that provides an example of the key messages to convey when counselors start PrEP education: “We understand that discussions surrounding health, particularly HIV, can be sensitive and possibly triggering due to various life experiences. Medicine exists to help prevent HIV, but many people are not aware of it. It is called PrEP, and it can be administered either as a daily pill or potentially as a long-acting injectable. Since some people with substance use disorders can have higher risk of HIV exposure, we routinely share information about PrEP with all our clients. People who do not have ongoing risks may have friends or family members with whom they can share this information. Whether you decide this information about PrEP is relevant for your situation or perhaps is useful for someone you know, we want to ensure that you have the access and the autonomy to make that decision.”

Adapting this approach ensures that all clients engaging in substance use counseling, regardless of their sociodemographic characteristics and life experiences, have equal access to PrEP education. Providing PrEP education for all recipients of substance use treatment services can reduce both provider and client discomfort associated with real and/or perceived assumptions about sexual orientation, gender, race, drug use, or sexual behaviors [34]. Given the stigma associated with injection drug use and queer relationships, some clients may not feel comfortable sharing personal information about their drug use and/or sexual behaviors. Thus, making PrEP education routine for all clients within a trauma-informed care helps foster a trusting and safe environment. It minimizes the risk of stereotyping marginalized groups or making assumptions about risk solely based upon clients’ sociodemographic characteristics and life experiences. Namely, discussion and provision of information, wherein everyone receives information as part of agency policy, becomes normalized. Additionally, counselors can help destigmatize and humanize PrEP uptake when they provide clients with PrEP education. For instance, some individuals may take daily birth control pills to prevent pregnancy. Likewise, individuals with type 2 diabetes may take daily medication to manage their blood glucose (i.e., sugar) levels. For clients with vulnerability to acquiring HIV, PrEP helps prevent HIV acquisition. With the recent advancements in PrEP, there is a long-acting injectable form of PrEP that has become a viable option for people with challenges in consistently taking daily medication.

In PrEP education, an open dialogue clarifying the purpose and benefit of PrEP administration can increase the likelihood that clients utilize the provided PrEP referral. For example, counselors may indicate that the use of PrEP can reduce the risk of sexually acquired HIV so that their clients can experience less anxiety and have more enjoyable sex if their partners have confirmed or suspected HIV acquisition. Substance use counselors should also inform clients about the primary goal of PrEP administration, which is to prevent people from HIV acquisition so that they may continue enjoying their daily activities and lives.

While there are disparities in HIV incidence across groups, not all members within a group are at equal risk of exposure to HIV [7,53,54]. Clients from lower HIV incident groups may have a higher individual need for PrEP that could go unidentified if they feel judged and/or unsafe to disclose their needs and decline PrEP education. When coupled with a strong working alliance, counselors can also encourage those seeking substance use treatment to share PrEP information with others, which will help to expand educational opportunities and provide an option for clients who may feel more comfortable asking questions in the context of helping others, rather than disclosing a personal need. When universal PrEP education is provided, a growing number of clients may express interest in learning more about PrEP, utilizing it, and being open to improving their HIV health literacy. A trauma-informed approach can be extended to guide counselors in facilitating PrEP access, preparing clients for a PrEP referral, and encouraging PrEP uptake and adherence.

### 5.3. Facilitating PrEP Access

Given that access to PrEP is a key component of HIV health literacy, substance use counselors play a pivotal role in connecting clients to services that meet their level of care and needs. When providing universal PrEP education, counselors can demystify the process of accessing PrEP by sharing clear, non-stigmatizing information about what to expect from medical providers specializing in HIV prevention. The medical setting can often be a space of vulnerability; therefore, counselors should make this process as transparent and supportive as possible to mitigate anxiety surrounding assessments and other procedures related to PrEP conducted by medical providers. Given research indicating discomfort across a range of professions as well as the practical challenge of the time needed to complete an in-depth sexual history [55,56,57,58,59], new PrEP guidelines recommend routine brief assessments (e.g., 1 to 6 questions) limited to only identifying key information associated with HIV acquisition risk among sexually active clients and persons who inject drugs. For details about the assessment process, we refer the reader to Appendix A of this article.

### 5.4. Preparing Clients for PrEP Referral

When clients indicate an interest in or need for PrEP, substance use counselors play an integral role in elucidating the details of PrEP and preparing clients for PrEP referral through a trauma-informed approach. For example, counselors can present clients with key information about PrEP administration. Being mindful of how trauma can impact a client’s experience in navigating healthcare, substance use counselors can help clients relieve the stress and anxiety associated with the process of PrEP referral and initiation by explaining what to expect, and when possible, providing referrals to PrEP providers who also provide trauma-informed care.

When explaining expectations, substance use counselors should explicitly share the following information with their clients to adequately prepare them for a PrEP referral. Prior to PrEP initiation, PrEP providers determine clients’ HIV status and the status of other infections (e.g., STIs, viral hepatitis), and assess their renal function. When initiating the provision of PrEP, providers provide medication regimens and relevant education to ensure the safety and effectiveness of, and adherence to PrEP therapy. Further, substance use counselors should offer clients ongoing support and prevention services to minimize clients’ exposure to HIV, even if they are on PrEP. Substance use counselors should make sure clients know to expect that PrEP providers will also likely encourage them to continue to use condoms during sexual activity and sterile injection equipment if they inject drugs while they are on PrEP. Clients should also be made aware that providers will monitor several indicators, such as HIV infection and medication toxicities, to make corresponding changes in regimens to promote long-term health in clients.

It is noteworthy to underscore the fact that both decisions and circumstances can change. A client who may initially decline a PrEP referral may experience ambivalence surrounding whether to start the regimen, and throughout counseling may later elect to accept a PrEP referral. Additionally, factors that impact vulnerability to HIV acquisition may change, such that a client may later accept a previously declined PrEP referral, highlighting the importance of revisiting the subject as appropriate. Likewise, a client may elect to discontinue PrEP if they no longer find themselves experiencing increased vulnerability. In preparing clients for PrEP, it is important for counselors to share this understanding and explain that restarting a PrEP regimen after stopping is an option.

In addition to informing clients about this process, substance use counselors should inform their clients about the options of injectable and oral PrEP to promote HIV health literacy through increasing knowledge of PrEP utilization. Of note, the CDC currently does not recommend injectable PrEP for persons who inject drugs, so counselors must remain aware of specific PrEP options for their client populations when providing PrEP education.

#### 5.4.1. Long-Acting Injectable vs. Daily Oral PrEP

Some clients can choose long-acting injectable PrEP (i.e., the cabotegravir extended-release injectable suspension) over daily oral PrEP for HIV. PrEP requires high levels of adherence to be effective against HIV acquisition. Research suggests that PWSUD and people with severe mental illness are less likely to adhere to daily oral medication due to possible associated poor social determinants of health and mental health conditions [60,61]. As a result of nonadherence, they will receive limited protection from oral PrEP medication. Injectable PrEP, an FDA-approved prevention method, is an option for clients who have difficulty with adherent use of daily oral PrEP and/or have severe renal disease. Meanwhile, oral daily PrEP is still an option when clients prefer oral daily PrEP to injectable PrEP for the reasons previously discussed, such as cost, insurance coverage, or having a history of reactions, like hypersensitivity. Namely, clients may experience difficulties obtaining insurance coverage for injectable PrEP [62]. Thus, counselors should provide clients with information about both options, and their benefits and potential challenges.

As of the writing of this article, the FDA has approved two types of daily single-dose oral PrEP—F/TDF and F/TAF—for individuals vulnerable to HIV acquisition. Of note, F/TAF is currently not approved for PrEP use by individuals assigned female at birth; F/TDF (oral PrEP) should be prescribed for this population if oral PrEP is desired. When educating clients with interest in PrEP, counselors should indicate that same-day prescriptions are often an option.

#### 5.4.2. Same-Day PrEP Prescription

Same-day PrEP prescription can provide more equitable access to PrEP and timely protection to clients with greater susceptibility to HIV acquisition. Some clinics have infrastructures and have developed protocols to conduct initial evaluations and prescribe PrEP on the same day. For example, such clinics need to conduct point-of-care HIV blood testing, ideally with antigen/antibody tests, as opposed to oral fluid testing, to obtain same-day results. When same-day HIV and creatinine test results are unavailable, clinics need to draw blood for laboratory HIV testing. Clinics must provide rapid follow-up contact to inform clients of the testing results and, if required, to schedule care appointments. It is also essential for clinics to help uninsured/underinsured clients to enroll in health insurance or medical assistance programs. Moreover, clinics must have clinicians available to administer required medication and injections.

### 5.5. Encouraging PrEP Uptake and Adherence

Counselors, well-trained to use their counseling skills, can augment their practice by integrating a trauma-informed approach to conceptualize their clients’ needs and support PrEP adherence among their clients who are prescribed PrEP for better protection from HIV. For instance, counselors can use attending skills (e.g., active listening, empathy) to establish trust and rapport so that clients can feel safe and comfortable sharing barriers and concerns over PrEP adherence and traumas that impact their PrEP adherence. As a result, counselors will experience fewer challenges in administering a brief medication adherence question to their clients: “It can be difficult for many people to take a medicine every day. During last week, how many days have you not taken your medicine?” If daily adherence is not possible or practical for some clients, counselors may share information about PrEP on-demand (i.e., taking PrEP pills only when people are at risk for getting HIV), an approach recommended by many state departments of health, despite not being approved by the FDA or endorsed by the CDC currently [63]. Moreover, counselors should consider explaining how PrEP works to keep people from seroconverting to HIV, discussing the possible side effects of PrEP (we refer readers to additional resources available at https://www.cdc.gov/hiv/basics/prep/about-prep.html (accessed on 21 July 2022)), and providing techniques that help clients manage emotional difficulties, such as stress and stigma, which are associated with using PrEP and missing medication. Importantly, understanding how past and ongoing traumas may be impacting clients’ adherence is vital to helping clients resolve barriers. For example, if a client is fearful that they will be abused if their medication is discovered, it would be necessary for a counselor to help them process their emotions as well as problem solve the situation so that they can take their medication as required. Counselors should also help clients process psychosocial problems (e.g., mental health disorders, social support) that prevent PrEP adherence. Additionally, of importance is that counselors should foster and celebrate clients’ growth in knowledge, attitudes, and adherence to PrEP in order to reinforce success.

Moreover, counselors should integrate certain short/long term goals into their clients’ treatment plans to improve PrEP adherence. First, counselors can help clients gain interpersonal skills to enhance and expand their social support systems. Second, counselors are trained to address clients’ mental health issues, substance use disorders, and financial needs, which significantly predict their PrEP adherence. Lastly, counselors can help clients establish and maintain medication routines that fit their work and social schedules by setting up reminder systems (e.g., creating multiple reminders on smartphones, using stickers on laptops/fridges). With increased efforts and experience working with people in need of PrEP, substance use counselors will be well positioned to contribute to HIV prevention and health outcomes among PWSUD, who are disproportionately affected by the syndemic.

## 6. Directions for Future Research

Considering the importance of universal PrEP education as a pivotal tool for HIV prevention, future research on universal PrEP education is warranted. First, understanding the efficacy and adaptability of universal PrEP education in diverse cultural and sociopolitical contexts can offer deeper insights into its cross-cultural and global applicability. The incorporation of trauma-informed approaches in PrEP educational strategies also requires more empirical investigations into its role in promoting HIV health literacy. Second, researchers can explore the relationships between health disparities in universal PrEP education, focusing on the role of socioeconomic factors and intersectional identities in influencing education outcomes among PWSUD such as HIV health literacy. The role of technology in extending the reach of PrEP education, particularly through telehealth and AI-assisted personalization, should also be carefully assessed. Concurrently, an analysis of healthcare systems and/or policies that examines how PrEP education can be seamlessly incorporated into existing healthcare practice may provide a roadmap for large-scale implementation. Psychological factors including the impact of mental health conditions and trauma histories on the outcomes of PrEP education offer additional understanding of the effectiveness of PrEP education and receptivity of it among PWSUD with co-occurring disorders in substance use treatment settings. Furthermore, longitudinal studies that track long-term impact and cohort-specific effects can provide temporal insights into the durability and effectiveness of PrEP education, which is an important HIV health intervention. Researchers can conduct quasi-experimental studies to examine the real-world effectiveness of universal PrEP education to provide real-world evidence to further inform clinical practices and public health policies [64,65]. Lastly, qualitative studies can contribute to a richer understanding of how universal PrEP education increases HIV health literacy among diverse clients. Given the syndemic of HIV, SUDs, and mental disorders, it is vital that future research considers these interconnected domains to generate scientifically rigorous and practical findings. These research initiatives hold the promise of enhancing both the theoretical and empirical landscape of universal PrEP education, thereby contributing to ongoing efforts in reducing the burden of HIV and promoting clients’ well-being.

## 7. Conclusions

Substance use counselors play a crucial role in reaching people with increased vulnerability to HIV. PrEP offers an effective HIV prevention strategy for PWSUD. When provided by counselors with sufficient knowledge of HIV and PrEP, universal PrEP education can enhance HIV health literacy, inform health decisions, and help navigate barriers to HIV preventive care. Counselors’ confidential relationship with clients offers a unique opportunity to convey genuine concern for their health, provide essential education about HIV and PrEP, and connect them to preventative resources, which requires counselors to stay attentive to referral agencies within their geographic area. Through universal PrEP education, including discussions on HIV, preventative medicine, and social determinants of health, counselors can improve HIV health literacy, contributing to the broader public health goal of reducing HIV transmission and promoting health outcomes among PWSUD.

## Figures and Tables

**Figure A1:**
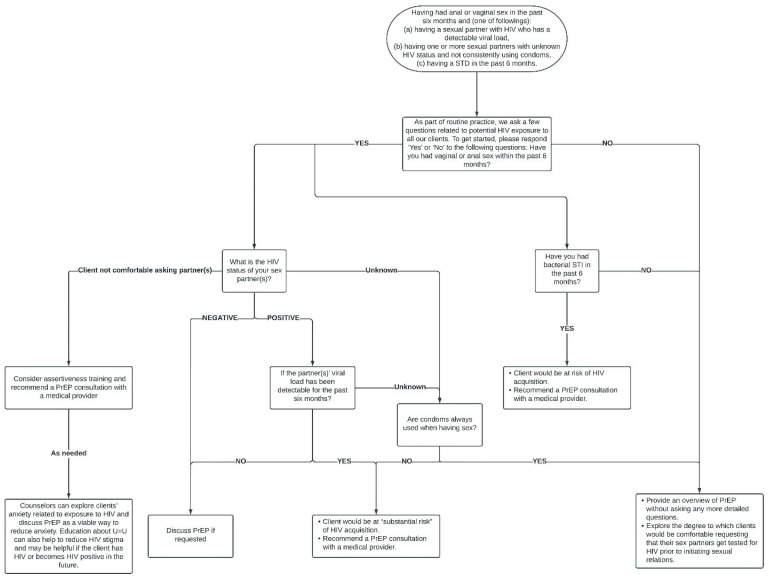
Identifying the need for PrEP referrals in sexually active clients.

**Figure A2:**
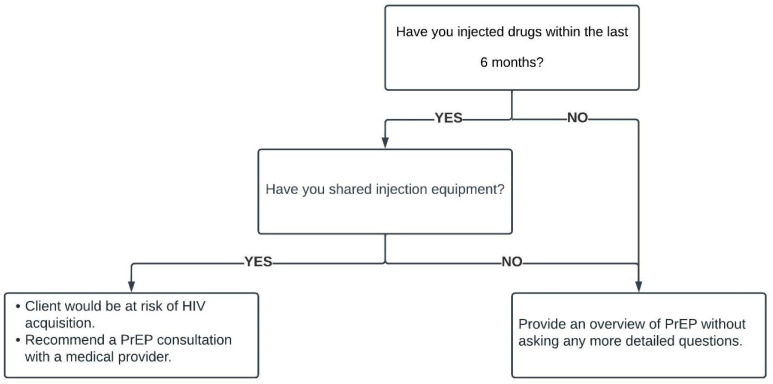
Identifying the need for PrEP referrals in people who inject drugs.

## Data Availability

Not applicable.

## Appendix A. Brief Assessment Recommended by the Latest PrEP Guidelines

Sexual-based indicators for PrEP include having had anal or vaginal sex in the past six months along with one or more of the following: (a) having a sexual partner living with HIV who has a detectable viral load, (b) having one or more sexual partners with unknown HIV status and not consistently using condoms, and (c) having an STD in the past 6 months. To begin an assessment of sexual HIV risk acquisition (see Figure A1), the counselor would first inform the client that it is routine practice to ask all clients a few questions to assess their risk of exposure to HIV. One way to initiate this process would be to say, “As part of routine practice, we ask a few questions related to potential HIV exposure to all our clients”. To get started, please respond “Yes” or “No” to the following questions: “Have you had vaginal or anal sex within the past 6 months?” Phrasing this question in a yes or no format and including vaginal and anal sex together avoids the discomfort of asking multiple questions or asking the client to specify specific practices. Counselors also do not need to ask about the gender or number of the clients’ sexual partners, as that may be stigmatizing for some and not essential for an initial assessment. If the client responds “no” to this question, then the counselor can provide an overview of PrEP without asking any more detailed questions.

Should clients indicate that they are sexually active by responding “yes” to the first question, then a follow-up question would be “Do you know the HIV status of your sex partner(s)?” When clients indicate their sex partners are living with HIV, the counselor would follow-up to see if the partner(s)’ viral load has been undetectable for the past six months. As needed, the counselor can explain the premise of “U = U” (known as Undetectable equals Untransmittable) [66], and the need for their sex partner to have a sustained, undetectable viral load (over the past 6 months) in order to prevent HIV transmission. If the partner’s viral load is undetectable, counselors should still provide clients with PrEP information when clients express any interest in PrEP or concerns over their partner’s risk of increasing HIV viral load outside their relationship. If the partner’s viral load is detectable, then the client would be at “substantial risk” of HIV acquisition and the counselor should recommend a PrEP consultation with a medical provider. Should a client show hesitancy or express discomfort about asking sex partners about HIV and viral load status, the counselor should consider assertiveness training and recommend a PrEP consultation with a medical provider. As needed, counselors can explore clients’ anxiety related to exposure to HIV and discuss PrEP as a viable way of reducing anxiety. Education about U=U can also help to reduce HIV stigma, and may be helpful if the client is living with HIV or acquires HIV in the future.

When the HIV status of sex partners is not known, the counselor will need to ask “Are condoms always used when having sex?” Even with consistent use of condoms, such a protective behavior is only associated with a 70% reduction in HIV transmission among men who have sex with men, and an 80% reduction of transmission from heterosexual intercourse [17,67]. Research findings indicate low rates of reported recent (within the last 30 days or at last sexual encounter) condom use among sexually active adults [68]. Rates of reported condom use decline further when research participants respond to questions about the consistent use of condoms over several months [69]. Therefore, regardless of consistent condom use, counselors should always explore PrEP initiation for clients whose sex partners’ HIV status is unknown. Additionally, as the counseling relationship develops, counselors should consider exploring the degree to which their clients would be comfortable requesting that their sex partners get tested for HIV prior to initiating sexual relations. Given varied life circumstances and relationship dynamics wherein clients’ safety and security may be at risk, counselors would need to use their clinical judgment to determine when and how to best address safe sex practices.

Another important question to include in the brief sexual history is whether the client has had a sexually transmitted infection (STI, e.g., chlamydia, gonorrhea, syphilis) during the past 6 months. A history of sexually transmitted infections has been found to be associated with potential HIV acquisition due to the likelihood of unprotected sex and unawareness of a partner’s sexual history [70]. Importantly, STI history is the greatest predictor of future HIV infection; this is not only due to behavioral factors, but also due to physical changes that increase vulnerability to HIV infection upon exposure [71]. Thus, counselors should explore PrEP initiation for these clients.

Injection drug use indicators of PrEP include sharing needles, syringes, or other equipment to inject drugs (e.g., gauze, torniquet, spoons) with others who are living with HIV or whose HIV status is unknown [7]. In addition to completing the brief sexual assessment of HIV acquisition, substance use counselors should also ask one to three questions to assess the risk of HIV acquisition from injection drug use (see Figure A2). The first question would be “Have you injected drugs within the last six months?” If the answer is “no”, and no other PrEP indicators arose from the brief sexual health history, the client would not be determined to have a substantial risk of HIV, and PrEP referral would not be indicated. If the answer is “yes”, then the counselor would need to ask if the client has shared injection equipment. If yes, then a referral for a PrEP consultation is recommended. Because of the stigma associated with reporting sexual behaviors and injection drug use, many clients will not feel comfortable disclosing this information [72,73,74]. As such, any clients who express an interest in PrEP should be provided with information and a referral for a PrEP consultation with a medical provider without requiring additional justification. When a PrEP referral is indicated, counselors need to be able to help their clients to be prepared for what to expect and support them through the decision-making process, including supporting their transition to PrEP use, as needed.
